# Synchronous Disease Kinetics in a Murine Model for Enterohemorrhagic *E. coli* Infection Using Food-Borne Inoculation

**DOI:** 10.3389/fcimb.2016.00138

**Published:** 2016-11-03

**Authors:** Laurice J. Flowers, Elsa N. Bou Ghanem, John M. Leong

**Affiliations:** ^1^Molecular Biology and Microbiology, Sackler School of Graduate Biomedical Sciences, Tufts UniversityBoston, MA, USA; ^2^Department of Molecular Biology and Microbiology, Tufts University School of MedicineBoston, MA, USA

**Keywords:** *Citrobacter rodentium*, shiga toxin, hemolytic uremic syndrome, enterohemorrhagic *E. coli*, food-borne inoculation

## Abstract

Upon colonization of the intestinal epithelium, the attaching and effacing (AE) pathogen Enterohemorrhagic *Escherichia coli* (EHEC) effaces microvilli and forms pedestal-like structures beneath the adherent bacterium. The production of one of its virulence factors, the phage-encoded Shiga toxin (Stx) results in systemic disease, including the development of renal failure. Although EHEC does not productively infect conventional mice, EHEC infection can be modeled in mice utilizing a derivative of the natural murine AE pathogen *Citrobacter rodentium* (CR). Gavage of mice with CR(ΦStx_2dact_), a *C. rodentium* lysogenized by a phage encoding an Stx variant with high potency in mice, features AE lesion formation on intestinal epithelium and Stx-mediated systemic disease, including renal damage. This model is somewhat limited by mouse-to-mouse variation in the course of disease, with the time to severe morbidity (and required euthanasia) varying by as many as 5 days, a feature that limits pathological analysis at defined stages of disease. In the current study, we altered and optimized the preparation, dose, and mode of delivery of CR(ΦStx_2dact_), using food-borne route of infection to generate highly synchronous disease model. We found that food-borne inoculation of as few as 3 × 10^4^ CR(ΦStx_2dact_) resulted in productive colonization and severe systemic disease. Upon inoculation of 1 × 10^8^ bacteria, the majority of infected animals suffered weight loss beginning 5 days post-infection and all required euthanasia on day 6 or 7. This enhanced murine model for EHEC infection should facilitate characterization of the pathology associated with specific phases of Stx-mediated disease.

## Introduction

Enterohemorrhagic *E. coli* (EHEC) O157:H7 is a Gram-negative bacterium and a causative agent of intestinal and systemic disease (Wadolkowski et al., 1990a; Robinson et al., 2006; Mohawk and O'Brien, 2011; Melton-Celsa et al., 2012; Kaper and O'Brien, 2014). Humans are infected by this organism through contaminated food or water. EHEC is a member of the family of pathogenic bacteria known as attaching and effacing pathogens (AE pathogens), which include enteropathogenic *E. coli* (EPEC) and *Citrobacter rodentium* (CR). These organisms are capable of triggering localized actin assembly on epithelial cells beneath bound bacteria, forming pedestal-like structures (Brady et al., 2007; Vingadassalom et al., 2009; Lai et al., 2013). EHEC, however, is unique because it produces a potent phage-encoded cytotoxin termed Shiga-toxin (Stx) that is the major agent that causes the tissue damage during EHEC infection (Wadolkowski et al., 1990a,b; Proulx et al., 2001; Thorpe et al., 2001; Robinson et al., 2006; Obrig, 2010; Melton-Celsa et al., 2012). Upon establishment of intestinal colonization, Stx promotes intestinal damage resulting in bloody diarrhea. The toxin subsequently translocates across the colonic epithelium into the bloodstream, where it targets distal tissues, including the microvasculature of the kidney, which expresses high levels of the Stx receptor, globotriaosylceramide (Gb3) (Obrig et al., 1993; Obata et al., 2008; Obrig, 2010; Melton-Celsa et al., 2012; Bunger et al., 2013). Hence, EHEC infection exhibits distinct clinical phases such as non-bloody diarrhea, followed by bloody diarrhea, and systemic disease, the latter manifested most commonly by hemolytic uremic syndrome (HUS), the triad of hemolytic anemia, thromobocytopenia and renal failure (Keepers et al., 2006; Obrig, 2010; Melton-Celsa et al., 2012; Davis et al., 2014; Melton-Celsa and O'Brien, 2014; Freedman et al., 2016).

Mice are highly sensitive to Stx, which can cause renal tubular damage following IP injection (Keepers et al., 2006; Mohawk and O'Brien, 2011; Mallick et al., 2012). However, EHEC does not efficiently infect conventional mice. Furthermore, although germ-free and antibiotic-treated mice exhibit susceptibility to EHEC infection, these models are not useful for studying EHEC pathogenesis factors, as strains that lack colonization factors required for AE lesion formation are not required for virulence in these models (Wadolkowski et al., 1990a; Eaton et al., 2008; Kamada et al., 2012). To address these limitations, an alternative model, using conventional mice, was developed. The native murine AE pathogen *C. rodentium* (CR), which like EHEC forms attaching and effacing lesions, was lysogenized with phage Φ1720a-02, isolated from an Stx-producing *E. coli* strain (Mallick et al., 2012, 2014). This phage encodes and produces Stx variant Stx2dact, which is activated ~18-fold by intestinal mucus (Mallick et al., 2012; Bunger et al., 2013), resulting in high potency in mice (Teel et al., 2002; Bunger et al., 2013). The oral gavage of mice with a high (5 × 10^9^ CFU) dose of an overnight culture of CR(Φ1720a-02), herein referred to as “CR(ΦStx_2dact_)” for simplicity, recapitulates many of the features of human EHEC infection in an Stx_2dact_-dependent manner, including colitis, renal damage, weight loss, and systemic injury.

Notably, a major limitation of this model is that disease kinetics are highly variable, with the duration of infection before mice require euthanasia varying from 4 to 9 days (Mallick et al., 2012). Thus, the unpredictable evolution of disease in this model precludes a careful characterization of pathology associated with distinct phases of infection (e.g., intestinal colonization, toxin dissemination, and renal failure). In the current study, we describe a method of preparation and delivery of the inoculum that results in a highly synchronous disease course, which should facilitate investigation of successive stages of Stx-mediated disease, and may more closely model the course of oral infection by an AE pathogen.

## Materials and methods

### Strains and inoculum preparation

Two days prior to infection, CR(ΦStx_2dact_) and CR(ΦStx_2dact_::*kan*^R^), described previously (Mallick et al., 2012, 2014), were streaked from frozen glycerol stock onto LB agar containing the appropriate antibiotic (i.e., 10 μg/ml chloramphenicol for CR(ΦStx_2dact_), and 10 μg/ml chloramphenicol and 25 μg/ml kanamycin CR(ΦStx_2dact_::*kan*^R^) for single colonies. The following day, a single colony was inoculated into 40 ml LB broth (without antibiotic) in a 50 ml conical tube (VWR, catalog number 89039-662) and incubated at 37°C with 5% CO_2_, without shaking but with the cap loosened to allow for gas exchange. Cultures were incubated typically for 10–12 h, to achieve an OD_600_ ~ 0.6–0.7. The culture was centrifuged at 10294 × g for 12 min at room temperature. The bacterial pellet was resuspended in 500 μl of Dulbecco's Phosphate-buffered saline DPBS (Life technologies, catalog number 14190-144) and then centrifuged at room temperature at 5226 × g for 2 min. Bacterial pellets were resuspended in 60 μl of DPBS. Six micro liter of a resuspended inoculum, encompassing ~ 1 × 10^8^ colony-forming units (cfu), was carefully pipetted onto a ~35 mg piece of Teklad 2918 irradiated rodent chow, visually ensuring that the inoculum was fully absorbed into the food (~1 min). For smaller inoculums, overnight cultures were appropriately diluted in PBS and into the food as described above. To titer the dose, a sample of the rodent chow inoculated in parallel was resuspended in 1 ml of DPBS, vortexed to disperse chow and bacteria, serially diluted, and plated on LB agar containing the appropriate antibiotic (i.e., 10 μg/ml chloramphenicol for CR(ΦStx_2dact_), and 10 μg/ml chloramphenicol and 25 μg/ml kanamycin CR(ΦStx_2dact_::*kan*^R^).

### Infection of mice

All procedures involving live animals were pre-approved by Tufts University Institutional Animal Care and Use Committee (IACUC). Five to six week old C57BL/6 mice were purchased from Jackson Laboratory and housed for 1–2 weeks in the animal facility at Tufts University School of Medicine prior to the start of infection. One day prior to infection, mice were weighed, cohoused on raised wire flooring to prevent coprophagy (Thoren caging systems: CC90F01), and food-restricted for 12 h. After food restriction, each mouse was individually placed into an empty cage and presented with a ~35 mg piece of inoculated rodent chow. After each mouse consumed the entire food inoculum, mice were weighed and returned to the original cage (without raised wire flooring) with access to unlimited food and water. For dose-response studies, mice were returned to caging with raised wire flooring to prevent coprophagy for the duration of the infection. Infection was followed by monitoring of CR(ΦStx_2dact_) or CR(ΦStx_2dact_::*kan*^R^) titers in the feces, as follows. Fecal samples, were weighed, suspended in DPBS at a volume of 10 μl per 1 mg of stool, and suspensions were serially diluted and plated on agar containing the appropriate antibiotic (10 μg/ml chloramphenicol for CR(ΦStx_2dact_), and 10 μg/ml chloramphenicol and 25 μg/ml kanamycin CR(ΦStx_2dact_::*kan*^R^). Mice were weighed and observed for clinical distress (ruffled fur, paucity of movement, and shivering) each day. Mice with greater than 12% body weight loss with or without signs of distress, or 10% body weight loss with signs of distress, were euthanized by CO_2_ inhalation followed by cervical dislocation. In addition, in some experiments, colonic and luminal colonization was measured by sacrificing mice at day 3, 4, or 5 post-infection. Each colon was dissected and stool contents were removed by gently pushing the stool through the colon with the smooth edge of a scalpel. The colonic luminal contents were collected by gently flushing the colons with 1 ml of DPBS. Colonic tissues were homogenized in 500 μl of DPBS using a Fisher Scientific PowerGen 125 Homogenizer. To determine the titer of CR(ΦStx_2dact_), colonic homogenates and luminal wash were serially diluted and plated on LB agar containing the appropriate antibiotic, as described above. For each weight loss experiment, the data passed the KS normality test.

## Results

### Inoculation of mice by feeding CR(ΦStx_2dact_) results in efficient colonization

The growth conditions of the inoculum are likely to influence the course of infection and disease. For example, host-adapted *C. rodentium* present in the feces of infected mice are in a hyper-infectious state, i.e., highly transmissible and capable of accelerated colonic colonization, compared to *C. rodentium* grown in LB (Wiles et al., 2005). EHEC grown in gnotobiotic piglets also exhibit enhanced virulence-related phenotypes (Brady et al., 2011). Thus, in considering ways to develop a consistent and predictable CR(ΦStx_2dact_) infection model, we first revisited the preparation of the inoculum. The previous described CR(ΦStx_2dact_) infection model, utilized bacteria that had been grown overnight to stationary phase (Mallick et al., 2012), a growth phase associated with global physiologic changes (Battesti et al., 2011), some of which alter infectivity and/or virulence (Molofsky and Swanson, 2004; Sonenshein, 2005). In addition, the CR(ΦStx_2dact_) cultures had previously been shaken, which facilitates aerobic growth and is predicted to sensitize *C. rodentium* to acid killing (Smith and Bhagwat, 2013). Hence, in our current study, we grew CR(ΦStx_2dact_) in a 5% CO2 atmosphere without shaking, a condition predicted to temper aerobic growth. These cultures were grown to an OD600 = ~0.6–0.7.

**Figure 1:**
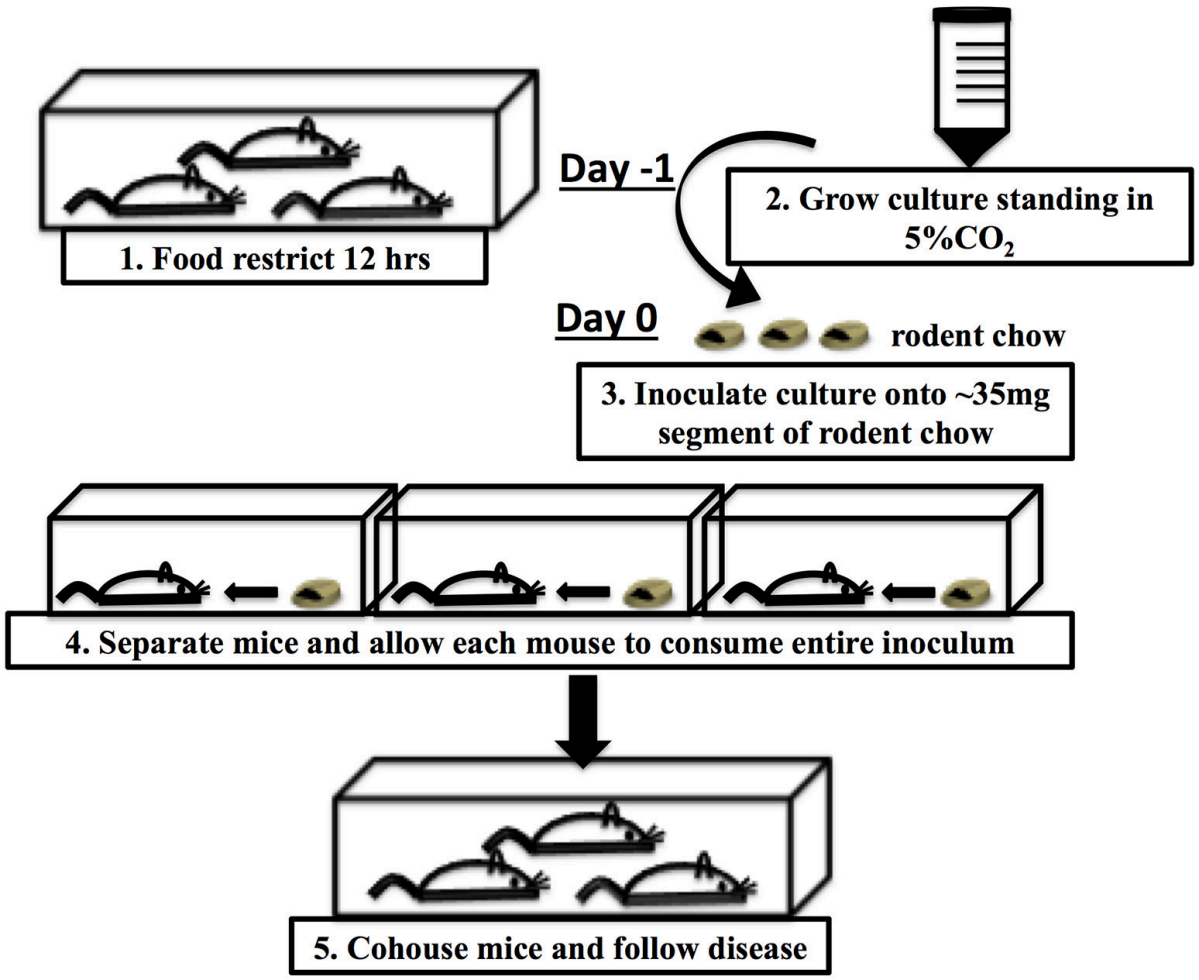
**Schematic of food-borne inoculation of mice**.

In the previously described CR(ΦStx_2dact_) model (Mallick et al., 2012), bacteria were delivered to the intestinal tract by oral gavage, a method that not only risks aspiration and esophageal and/or gastric puncture, but also induces a host stress response that might alter the course of infection (Hoggatt et al., 2010; Walker et al., 2012). Methods for intestinal delivery of pharmacological agents by feeding have been established that minimize this stress response (Walker et al., 2012). Further, delivery of *Listeria monocytogenes* in food rather than by gavage altered the fate of bacteria in the host and the virulence factors required for infection. (Bou Ghanem et al., 2012). Hence, 6–8 week old C57BL/6J mice were inoculated by ingestion of 1 × 10^8^ CFU of CR(ΦStx_2dact_) absorbed into a small (~35 mg) piece of rodent chow (Figure 1 and Materials and Methods).

**Figure 2:**
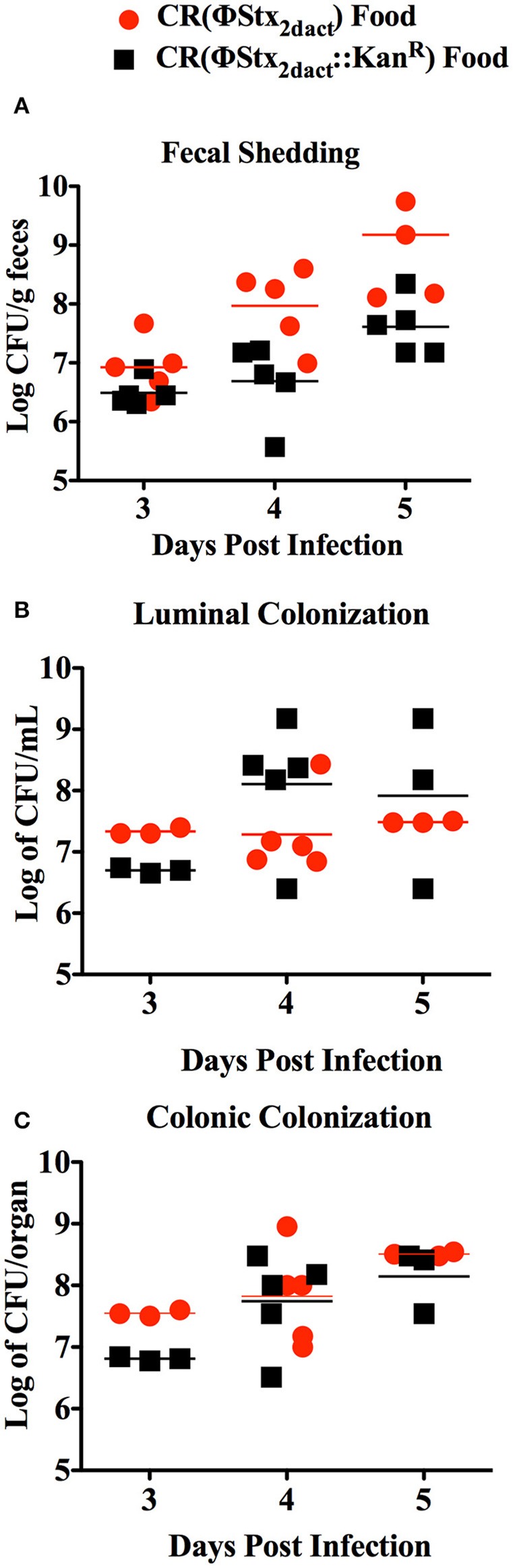
**Inoculation of mice by feeding CR(ΦStx_2dact_) results in efficient colonization**. Six-week old female C57/BL6 mice were inoculated with 1 × 10^8^ CR(ΦStx_2dact_) or CR(ΦStx_2dact_::*kan*^R^) by feeding and viable counts of feces **(A)**, colonic homogenates **(B)**, or luminal washings **(C)** were determined by plating. Each point represents an individual mouse and each line represents the geometric mean.

To determine if inoculation of mice with CR(ΦStx_2dact_) in this manner resulted in efficient intestinal colonization, we used fecal shedding CR(ΦStx_2dact_) as a non-terminal indicator of colonic colonization: on successive days following infection, we plated for viable CR(ΦStx_2dact_) on selective media. High levels of fecal shedding, i.e., ~10^7^ cfu/g of feces, were observed on day 3 post-infection, increasing to >10^8^ cfu/g of feces on days 4 and 5 post-infection (Figure 2A). High levels of colonization were also observed in colonic luminal washes and colonic homogenates following sacrifice. These titers of CR(ΦStx_2dact_) were roughly equal to the peak fecal shedding, colonic colonization, and luminal washes previously reported (Mallick et al., 2012, 2014) by intra-gastric inoculation. These data indicate that ingestion of food inoculated with mid-log phase bacteria grown without shaking results in efficient intestinal colonization.

The production of Stx by *C. rodentium* has been associated with slightly higher levels of intestinal colonization following inoculation by oral gavage (Mallick et al., 2012, 2014). Therefore, we also infected mice with CR(ΦStx_2dact_::*kan*^R^), a CR(ΦStx_2dact_) mutant that does not produce Stx. Fecal shedding of CR(ΦStx_2dact_::*kan*^R^) was indistinguishable from that of CR(ΦStx_2dact_) (Figure 2A), and CR(ΦStx_2dact_::*kan*^R^) titers were similar to those of CR(ΦStx_2dact_) in luminal washes and colonic homogenates. Thus, at least for this small sample size (*n* = 5), Stx did not detectably promote colonization in this model (Figure 2).

### Food-inoculation of mice with as few as 3 × 10^4^ CR(ΦStx_2dact_) results in colonization, disease, and mortality

**Figure 3:**
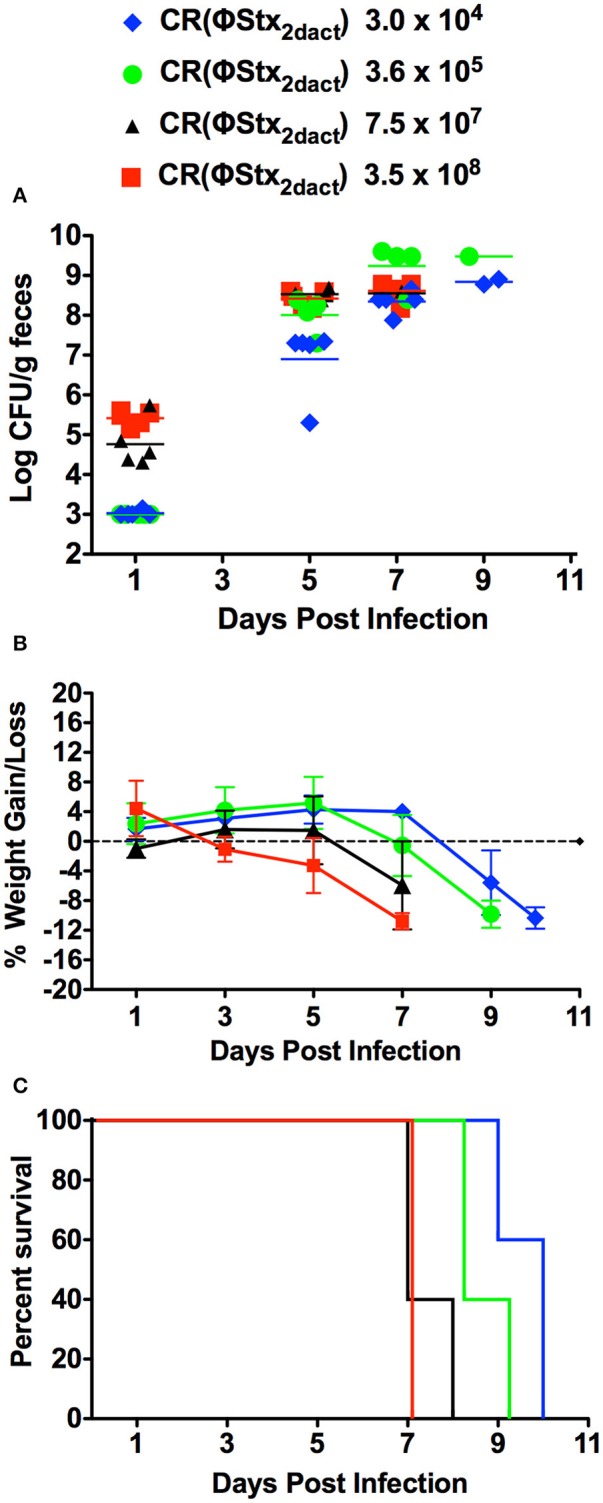
**Food-borne inoculation of mice with as few as 3 × 10^4^ CR(ΦStx_2dact_) results in colonization, disease and mortality**. Groups of five mice were food-inoculated with the indicated dose of CR(ΦStx_2dact_) and fecal shedding **(A)**, body weight as percent change from that prior to infection **(B)** and percent survival **(C)** were determined as described in Materials and Methods. In **(A)**, each point represents an individual mouse and each line represents the geometric mean. For all panels, shown is a representative of two independent experiments.

Mice are typically gavaged with 10^8^ *C. rodentium* to initiate productive infection. To determine if inoculation of C57/BL6J mice with lower doses of CR(ΦStx_2dact_) by our feeding model would result in productive infection and disease, we fed groups of 10 mice doses of CR(ΦStx_2dact_) spanning four orders of magnitude, from 3.5 × 10^8^ down to 3 × 10^4^. To avoid coprophagy, which due to the ingestion of bacteria shed in feces would confound accurate quantitation of the oral dose, the mice were housed on elevated wire floors for the duration of the infection. Fecal shedding of CR(ΦStx_2dact_) revealed that although the kinetics of intestinal colonization appeared to be slightly delayed in mice receiving the lowest dose, all groups of mice were colonized by day 5 post-infection and reached >10^8^/g stool by day 7 (Figure 3A). Similarly, all groups of mice lost weight, the kinetics of which varied with dose: the first indication of significant weight loss occurred on post-infection day 7 for the group of mice infected with 7.5 × 10^7^ or 3.5 × 10^8^ CR(ΦStx_2dact_), day 8 for the group infected with 3.6 × 10^6^, and day 9 for the group infected with 3.0 × 10^4^ (Figure 3B). Finally, all groups of mice became moribund and required euthanasia (Figure 3C). Consistent with the trends observed for the kinetics of intestinal colonization and weight loss, mice infected with lower doses exhibited a somewhat longer interval between inoculation and required euthanasia: by day 7 post-infection, the group of mice infected with 3.5 × 10^8^ CR(ΦStx_2dact_) were all euthanized, whereas for groups of mice infected with 7.5 × 10^7^, 3.6 × 10^5^, and 3.0 × 10^4^ bacteria, the day at which 100% were euthanized was day 8, 9, and 10, respectively. We conclude that feeding of C57/BL6J mice with as few as 3.0 × 10^4^ CR(ΦStx_2dact_) results in productive and lethal infection.

### Mice inoculated with CR(ΦStx_2dact_) by feeding suffer synchronous disease and mortality

**Figure 4:**
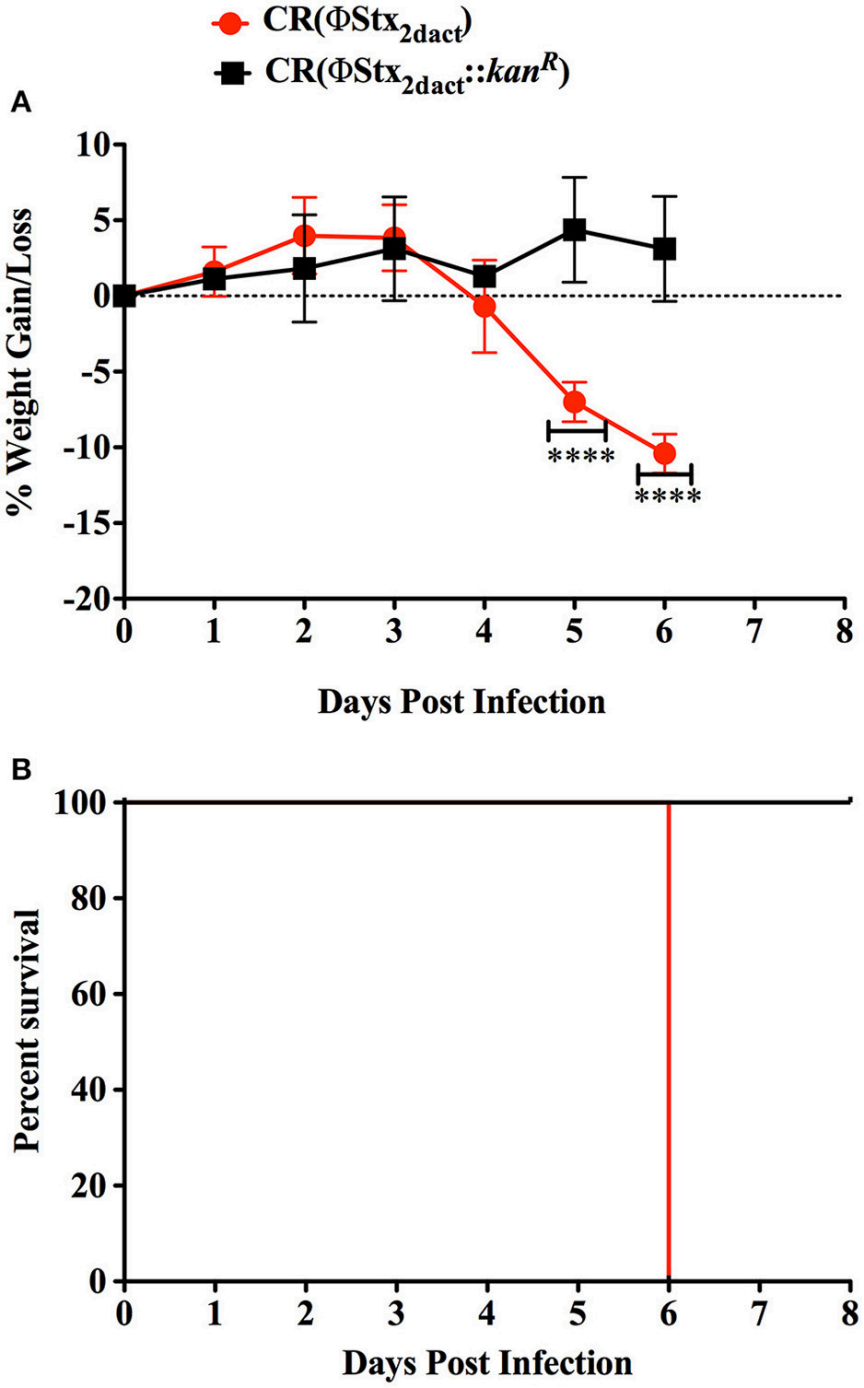
**Inoculation with CR(ΦStx_2dact_) by feeding results in synchronous disease and mortality**. Groups of five mice were inoculated with 1 × 10^8^ CR(ΦStx_2dact_) or CR(ΦStx_2dact_::*kan*^R^), and mean (± SD) body weight as percent change from that prior to infection **(A)** and percent survival were monitored daily **(B)**. ^****^Indicates *p* > 0.0001 by 2-way Anova followed by Bonferroni post-test. Shown is a representative of the four independent experiments that are described in Table 1.

Previous work revealed that the kinetics of disease following gavage inoculation with stationary phase CR(ΦStx_2dact_) varied widely, e.g., resulting in required euthanasia as early as 4 days and as late as 9 days post-infection within a given experiment (Mallick et al., 2012). In contrast, the trend of delayed colonization and disease kinetics observed for mice given different doses of CR(ΦStx_2dact_) by feeding suggested that the time course of disease in this model may be more consistent than by oral gavage. To carefully assess the time course of disease, in multiple experiments mice were weighed daily after inoculation with mouse chow containing the relatively high dose of 1 × 10^8^ CR(ΦStx_2dact_), or, as a control to evaluate Stx-dependent disease, CR(ΦStx_2dact_::*kan*^R^). (For simplicity of the protocol, and in contrast to the dose-response studies described above, mice were not housed on wire floors after inoculation, raising the possibility that the dose may be somewhat higher due to coprophagy.) In our first experiment, all five mice infected with CR(ΦStx_2dact_) crossed a threshold of 4.5% body weight loss on day 5 post infection, whereas mice infected with CR(ΦStx_2dact_::*kan*^R^) did not suffer weight loss (Figure 4A; Table 1, Experiment 1). In addition and in contrast to mice infected with the Stx-deficient strain, which uniformly survived infection, all mice infected with CR(ΦStx_2dact_) required euthanasia on day 6 (Figure 4; Table 1). To assess the reproducibility of the course of disease in this model, we repeated the experiment three times for a total of four independent experiments encompassing 20 mice (Table 1). Although the kinetics of weight loss and required euthanasia for Experiments 2 through 4 were not as uniform as in Experiment 1, cumulatively 70% of mice reached the threshold of >4.5% body weight loss on day 5 post-infection, and all mice required euthanasia on day 6 or 7 (Table 1). We conclude that this infection protocol, in contrast to the gavage model using stationary phase CR(ΦStx_2dact_), results in a highly synchronous course of disease.

**Table 1:** **Synchronous kinetics of disease following CR(ΦStx_2dact_) infection**.

	**Initial observance of >4.5% weight loss**				**Euthanasia required^a^**	
	**Day 3**	**Day 4**	**Day 5**	**Day 6**	**Day 6**	**Day 7**
Experiment 1^b^	–	–	5/5	–	5/5	–
Experiment 2	–	1/5	4/5	–	5/5	–
Experiment 3	–	–	4/4^c^	–	1/5	4/5
Experiment 4	3/5	1/5	–	1/5	–	5/5
Total	3 (15%)	2 (10%)	14 (70%)	1 (5%)	11 (55%)	9 (45%)

a See Materials and Methods for criteria for euthanasia.

b Depicted in Figure 4 and included here for ease of comparison with Experiments 2–4.

c Data for weight loss of one mouse was lost.

## Discussion

Serious life-threatening cases of EHEC infection evolve through successive phases of illness, beginning asymptomatically but followed by the development of non-bloody diarrhea, bloody diarrhea, and, in the most serious cases, culminating in HUS (Thorpe et al., 2001; Keepers et al., 2006; Obrig, 2010; Mohawk and O'Brien, 2011; Melton-Celsa et al., 2012; Davis et al., 2014; Freedman et al., 2016). As the success of a given clinical intervention may depend on the phase of illness, animal models of EHEC infection that permit analysis of specific phases of disease are invaluable to understanding the evolution of disease and developing successful therapeutic strategies. Murine infection by oral gavage with CR(ΦStx_2dact_) represents a useful EHEC infection model that features AE lesion formation, intestinal epithelial damage, and Stx-mediated renal damage (Davis et al., 2014). However, this model lacks the synchronicity of disease evolution, reflected by considerable variation in time to morbidity (Mallick et al., 2012), thus greatly complicating detailed investigation of stage-specific pathology.

In the current study, we altered two features of the existing CR(ΦStx_2dact_) model. First, because *C. rodentium* anaerobiosis is associated with enhanced acid-resistance and a transcriptomic profile more similar that of host-adapted, hyper-infectious bacteria (Smith and Bhagwat, 2013), we changed our culturing procedures by growing inocula without shaking. Studies with other enteric pathogens have revealed that the method of inoculum preparation can have dramatic effects on virulence in animal models (Clark et al., 1998; Brady et al., 2011). Second, we infected mice by feeding to avoid potential physical trauma. In addition, gavage is associated with host stress, reflected in an increase in heart rate, mean arterial pressure, and fecal corticosteroids (Hoggatt et al., 2010; Walker et al., 2012), factors that might contribute to mouse-to-mouse variability. Mice infected using this modified protocol were efficiently colonized even at doses as low as 3 × 10^4^ bacteria, as reflected by titers of CR(ΦStx_2dact_) in feces and colonic homogenates. (In fact, further infection studies suggest that food-borne inoculation of doses fewer than 10^4^ CR(ΦStx_2dact_), 2.35 × 10^3^ and 6 × 10^3^, also promotes colonization and disease; LJF, unpublished data). Although we did not perform a parallel dose-response study with gavage-delivered bacteria, this 3 × 10^4^ dose is three or four orders of magnitude lower than what has been typically used in gavage studies (Vallance et al., 2003; Tennant et al., 2008; Bergstrom et al., 2010; Diez et al., 2011; Kamada et al., 2012). In contrast to this study, a previous study with gavage-inoculated mice suggested that fecal shedding of CR(ΦStx_2dact_) was slightly higher than that of CR(ΦStx_2dact_::*kan*^R^) (Mallick et al., 2012, 2014). However, detection of a statistically significant difference in colonization required much larger numbers of mice (*n* ≥ 20) than utilized in this study.

Importantly, compared with the previously described gavage model, infection using our model produced relatively synchronous disease; when four independent experiments were analyzed together, 70% of mice reached a threshold of >4.5% body weight loss on day 5 post-infection, and all mice required euthanasia on day 6 or 7 post-infection. Given that oral gavage is by far the most common method of inoculation for models of intestinal pathogens, we speculate that the preparation and inoculation methods described here might be widely applied to improve the reliability and synchronicity of other infection models.

It is important to note that several aspects of human HUS are not faithfully replicated in mice. Although murine Stx-injection or EHEC infection models have been associated with increased renal (as well as systemic) cytokines, renal damage is typically limited to tubule pathology and lacks the characteristic glomerular damage associated with HUS (Mallick et al., 2012; Melton-Celsa et al., 2012). This difference has been postulated to be due to the paucity of Gb3, the Stx receptor, on murine podocytes and renal endothelial cells, whose counterparts in humans are important targets of Stx intoxication (Keepers et al., 2006). Genetic manipulation of mice to ectopically produce Gb3 on critical target cells, in combination with the improved CR(ΦStx_2dact_) infection protocol described here, might facilitate the systematic investigation of the evolution of Stx-mediated disease upon EHEC infection.

## Author contributions

LF: Designed and performed the experiments and wrote the paper. EB: Suggested critical parameters in design of experiments and edited paper. JL: Provided advice in design, performance of experiments and co-wrote paper.

### Conflict of interest statement

The authors declare that the research was conducted in the absence of any commercial or financial relationships that could be construed as a potential conflict of interest.
